# Solution structure of the transmembrane domain of the mouse erythropoietin receptor in detergent micelles

**DOI:** 10.1038/srep13586

**Published:** 2015-08-28

**Authors:** Qingxin Li, Ying Lei Wong, Michelle Yueqi Lee, Yan Li, CongBao Kang

**Affiliations:** 1 Institute of Chemical & Engineering Sciences, Agency for Science, Technology and Research (A*STAR), Singapore, Singapore; 2 Experimental Therapeutics Centre, Agency for Science, Technology and Research (A*STAR), Singapore, 138669 Singapore

## Abstract

Erythropoiesis is regulated by the erythropoietin receptor (EpoR) binding to its ligand. The transmembrane domain (TMD) and the juxtamembrane (JM) regions of the EpoR are important for signal transduction across the cell membrane. We report a solution NMR study of the mouse erythropoietin receptor (mEpoR) comprising the TMD and the JM regions reconstituted in dodecylphosphocholine (DPC) micelles. The TMD and the C-terminal JM region of the mEpoR are mainly α-helical, adopting a similar structure to those of the human EpoR. Residues from S216 to T219 in mEpoR form a short helix. Relaxation study demonstrates that the TMD of the mEpoR is rigid whilst the N-terminal region preceding the TMD is flexible. Fluorescence spectroscopy and sequence analysis indicate that the C-terminal JM region is exposed to the solvent. Helix wheel result shows that there is hydrophilic patch in the TMD of the mEpoR formed by residues S231, S238 and T242, and these residues might be important for the receptor dimerization.

Erythropoiesis is a process to produce red blood cells. It is a tightly regulated process, which is important to sustain the normal biological functions^1^. The hormone erythropoietin (EPO) is essential for proliferation and differentiation of red cell precursors through its receptor (EpoR)^2^. EPO binding to EpoR causes a conformation change, which can activate the Janus kinase 2 (JAK2) at its cytoplasmic side^3^. Activated JAK2 will phosphorylate several tyrosine residues in the cytoplasmic region of the EpoR to produce docking sites for Src-homology 2 (SH2) domain-containing proteins that are essential for the activation of the mitogen-activated protein kinase pathway^2^.

EpoR is a single-span transmembrane protein and belongs to the cytokine receptor superfamily^4^. Receptors within this family including growth hormone receptor (GHR), prolactin receptor (PR) and thrombopoietin receptor (TpoR) are key regulators of many processes. The EpoR is expressed in erythroid progenitors derived from bone marrow and several non-hematopoitic tissues^2^. Like other receptors such as GHR, EpoR was demonstrated to form an inactive dimer in the absence of its ligand^5^. The EpoR consists of an extracellular region, juxtamembrane (JM) regions, a transmembrane domain (TMD) and a cytoplasmic region (Fig. 1A). Structural study has shown that the extracellular region contains two fibronectin type II domains that can form a dimer in the absence of EPO^5^. Ligand binding to the extracellular domain causes conformational changes, which is necessary for the activation of JAK2^6^. The TMD and the JM regions of the EpoR are important for the receptor function. Constantinecu *et al.* showed that the TMD is sufficient for the ligand-independent dimerization^7^. The C-terminal JM region is important for JAK2 activation through a hydrophobic motif formed by several hydrophobic residues^8^. The cytoplasmic region of the EpoR is essential for JAK2 activation through two regions^9^.

The mechanism of ligand induced JAK2 activation was described in a recent study using GHR as a model^10^. In this study, GHR was shown to form a dimer *in vivo* through its TMD. The TM helices are parallel in the basal state and form a left-hand crossover state when the receptor binds to its ligand. The movement of the TMD helices results in the removal of the pseudokinase inhibitory domain of JAK2 to activate JAK2^10^. The receptor activation mechanism is through the function of TM helix dynamics in a lipid membrane and the EpoR may also be suitable for this model^11^. Based on the accumulated studies, it is obvious that the TMD of the EpoR is important for receptor function. The TMD of the EpoR are also shown to form dimers when it was reconstituted into detergent micelles^12^. We also conducted a structural study on the human EpoR (hEpoR) in dodecylphosphocholine (DPC) micelles. The hEpoR was demonstrated to be able to form dimers in micelles and its JM region formed a helix as predicted^13^.

The TMD of mouse EpoR (mEpoR) and hEpoR share very high sequence homology. It was demonstrated that mEpoR might have a higher binding affinity than hEpoR, which might be one of the reasons that mEpoR is more active than hEpoR^12^. To further understand the structure of the mEpoR, we present a nuclear magnetic resonance (NMR) study of the TMD and JM regions comprising residues 212–259 of the mouse EpoR. Our results show that mEpoR has a similar structure to hEpoR with the exception that its N-terminal region preceding the TMD contains a short helix due to lack of a proline residue. There is a hydrophilic patch formed by residues S231, S238 and T242 in the TMD. Our structural and dynamic information of the mEpoR will be useful to understand the role of the TMD and JM regions in signal transduction.

## Results

### Secondary structure of mEpoR

**Figure 1:**
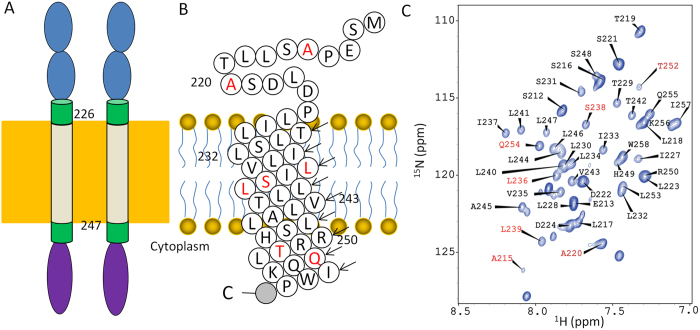
Topology of the mEpoR. (**A**) Diagram of the EpoR. The extracellular domain, JM regions, TMD and the cytoplasmic region are shown in blue, green, gray and purple, respectively. The TMD contains residues from L226 to L247. (**B**) Sequence topology of the mEpoR used in this study. Sphere highlighted in gray indicates the histidine tag. Residues highlighted in red are different from those in hEpoR. (**C**) Assignment of the ^1^H-^15^N-HSQC spectrum of the mEpoR. The peaks are labeled with residue name and sequence number. Residues that are different from human EpoR are highlighted in red.

**Figure 2:**
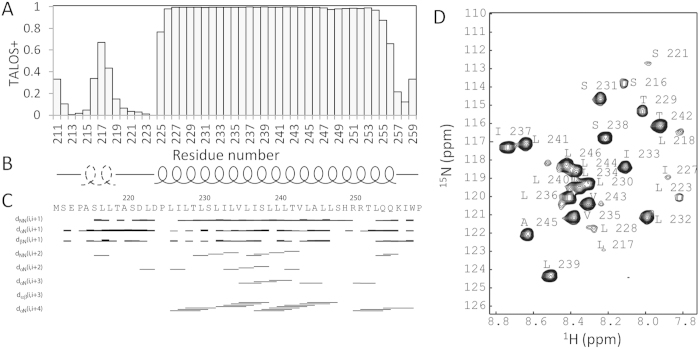
Secondary structure of the mEpoR. (**A**) TALOS+ analysis of mEpoR in DPC micelles. (**B**) Secondary structure of the mEpoR. The helix from the residues at the N-terminus is drawn with a dotted line. (**C**) NOE connectivity of mEpoR. The defined TM helix is characterized by the inter-proton NOE connectivity of Hα^i^ to H_N_^i+4^. C. ^1^H-^15^N-HSQC spectrum of the mEpoR in D_2_O. Residues exhibiting cross-peaks in the spectrum are considered to have helical structures.

To conduct structural and dynamic studies for the mEpoR (Fig. 1), the construct was purified in the presence of DPC micelles. The backbone assignment of the mEpoR in DPC micelles was achieved using conventional 3D experiments (Fig. 1C)^13^. The assignment has been deposited into the BioMagResBank under accession number 25396. The calculated chemical shift index from the assigned Cα chemical shifts indicated that the mEpoR has similar structure to the hEpoR^13^. Further secondary structural analysis of backbone chemical shifts using TALOS+ indicated that the TMD of the mEpoR forms a helix. Residues compassing S216 to T219 preceding the TMD have a tendency to form a short helix (Fig. 2A,B). Residue 220 of mEpoR is an alanine instead of a proline in hEpoR, which might favor the formation of this short helix. The sequential NOE connectivity result also confirmed the secondary structural elements in mEpoR (Fig. 2C). The TMD and its C terminal JM region of the mEpoR form a helix, which is similar to the hEpoR. H-D exchange experiment also suggested that residues in the TMD were protected from exchanges (Fig. 2D). Missing the assignment of the backbone amide and amide proton of R251 and lacking inter-proton NOE connectivity between R250 and its preceding residues suggested that residues R250 and R251 might have exchanges with the environment. TALOS+ analysis showed that residues from S216 to T219 have a tendency to be a helix, but the NOEs between Hα of residue *i* and HN of residue *i* + 3(4) were not observed. This might arise from the fact that this region is flexible (Fig. 2C).

### Backbone dynamics of the mEpoR in DPC micelles

**Figure 3:**
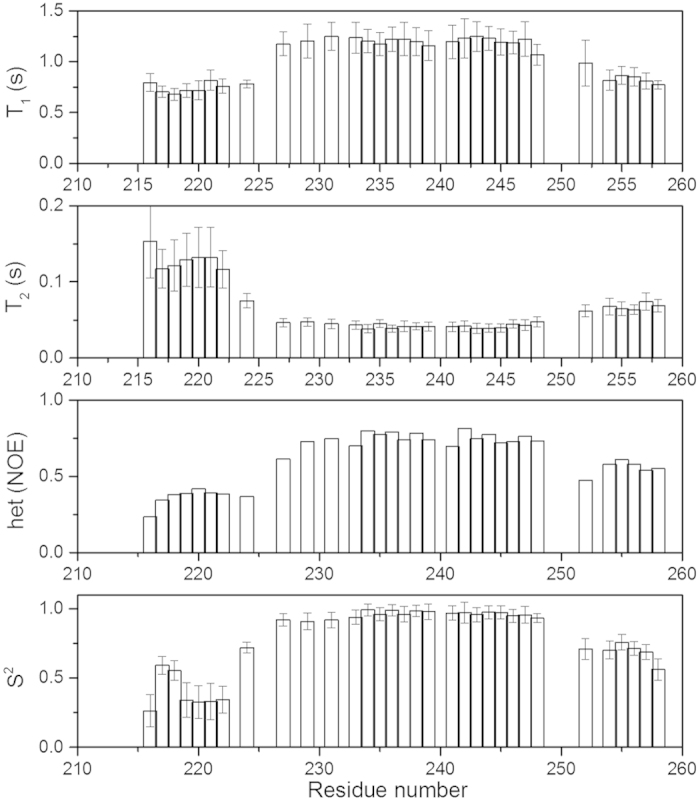
Backbone ^15^N relaxation dynamics of the mEpoR in DPC micelles. The ^15^N T_1_, T_2_, hetNOE and the order parameter (S^2^) are plotted as a function of residue number. The experiment was conducted on a Bruker magnet with a proton frequency of 600 MHz. The experiment was performed 313K as previously mentioned. The T_1_, T_2_ and hetNOE were determined using NMRView and the S^2^ was obtained using model-free software. Results for overlapped, unassigned and proline residues are not shown.

To understand the flexibility of the mEpoR in the picosecond-to-nanosecond timescale, the backbone T_1_, T_2_ and ^15^N-^1^H-heteronuclear NOE (hetNOE) experiments were measured at 600 MHz proton resonance frequencies (Fig. 3). High T_1_, hetNOE and low T_2_ values of the TMD suggested it is rigid in solution. The N-terminal region of the construct is dynamic characterized by low T_1_, hetNOE and high T_2_ values. For the C-terminal JM region compassing residues L253 to W258, it exhibited a hetNOE value close to 0.6. The generalized order parameter (S^2^) was obtained using a model-free approach (Fig. 3). Residues from 227 to 248 exhibited S^2^ values close to 0.85, suggesting that these residues are rigid in solution. Residues from 252 to 258 exhibited S^2^ values close to 0.7, suggesting that this region is not as rigid as the TMD. For the N-terminal region, most of the residues showed low S^2^ values and residues 217 and 218 exhibited slightly higher S^2^ values than other residues within this region, which might arise from the fact that these two residues are in the short helix (Fig. 3).

**Figure 4:**
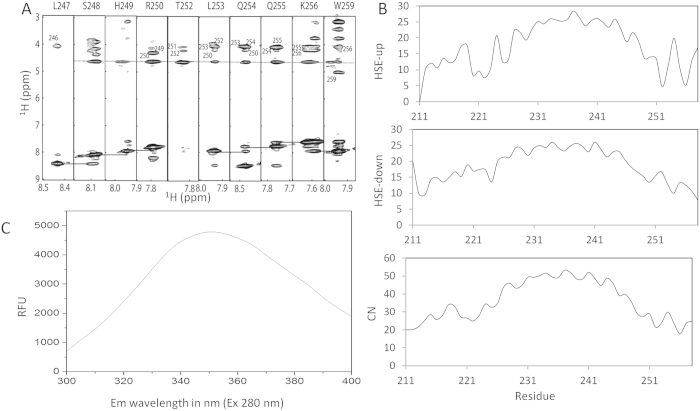
Exposure of the C-terminal JM region to the solvent. (**A**) NOE between residues in the JM region and water. The slice from a 3D-HSQC-NOESY was plotted. The NOE between amide proton and water protons is indicated as a solid line. The assignments of the Hα atoms are labeled with sequence number. Sequential connection of amide protons are shown with solid lines. (**B**) Solvent exposure analysis using protein sequence. HSE analysis was carried out and the resulting HSE values and CN are plotted against residue number. (**C**) Fluorescence spectroscopy of the mEpoR in DPC micelles. The experiment was conducted as described in Materials and Methods.

To further understand whether the C-terminal JM region is exposed to the solvent, we analyzed our NOE data. It is clear that the amide protons from hydrophilic residues compassing 250 to 257 exhibited NOEs with water protons, demonstrating that these residues were not buried in DPC micelles (Fig. 4A). Interestingly, amide protons of the three conserved hydrophobic residues including L253, I257 and W258 showed no NOE or weak NOEs with water protons, which indicated that these residues may interact with micelles or having exchanges. We then performed bioinformatics analysis of the construct using a half-sphere exposure (HSE) algorithm^14^. In the HSE analysis, a residue is considered as a sphere with two half parts. HES-up corresponds to the direction of the chain side of a residue and HES-down corresponds to the direction of the opposite side. This method also predicts the contact number (CN) that is a measurement of a residue burial in proteins. The HSE-up showed that most residues in the TMD and few residues in both N- and C-termini were protected from exposure. Taking the HES-down and CN results together, the N- and C-termini of the constructs are exposed to the solvent (Fig. 4B). Although this bioinformatics study might not suitable for mEpoR, the information obtained suggested that the C-terminal JM region is exposed to the solvent. There is only one tryptophan residue present (W258) in the protein sequence. Tryptophan fluorescence experiment was carried out. In the fluorescence spectrum, the emission maximum of the mEpoR in DPC micelles was 350 nm (Fig. 4C), indicating that W258 is exposed to the solvent because the emission maximum will be shifted to a lower wavelength if the tryptophan residue is buried in a hydrophobic environment.

### Structure of the mEpoR in DPC micelles

**Figure 5:**
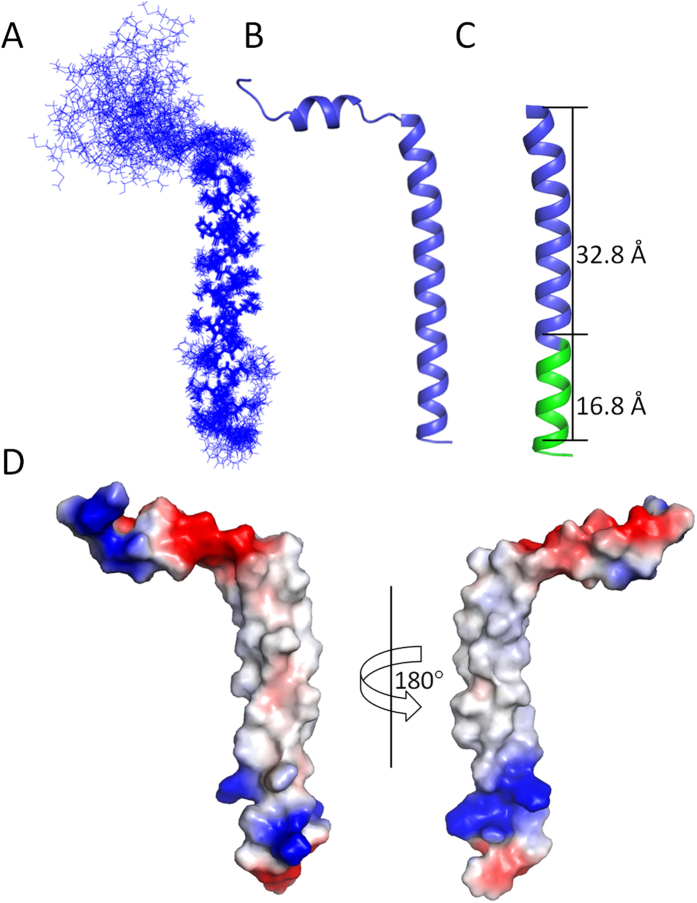
Structure of the mEpoR in DPC micelles. (**A**) Twenty superimposed mEpoR structures. All the side chains of the residues are shown. (**B**) One model of the mEpoR with lowest energy. The N-terminus contains a short helix and the TMD and JM form an extended helix. (**C**) Structure of the mEpoR. The height of the TMD helix is shown. For clarity, the N-terminus of the construct is not shown. (**D**) Color-coded electrostatic surface potential for the mEpoR. The positive, negative and hydrophobic surfaces are shown in blue, red and white, respectively. All the pictures are drawn using PyMOL (www.pymol.org).

Structures of the mEpoR were solved using NOE restraints derived from NOE experiments, dihedral angle restraints derived from TALOS+ and hydrogen bond restraints based on H-D exchange experiment. The calculated 20 lowest energy structures do not have any distance or dihedral angles violations that exceed 0.5 Å and 5°, respectively. The structural ensemble has been deposited in PDB under accession number 2MXB. Table 1 shows the statistics of the determined mEpoR structures. These structures show an extended transmembrane helix formed by residues from 225–257 with backbone r.m.s.d. of 0.60 ± 0.21 Å. The length of the TMD is 32.8 Å and the C-terminal JM region is helical. Although the N-terminus of the mEpoR is flexible, there is a short helix formed by residues from S216 to S221 (Fig. 5B). Electrostatic surface potential analysis of the mEpoR indicated that residues from 226 to 247 form a hydrophobic surface, which is not surprising for the TMD (Fig. 5C). The N-terminal region of mEpoR contains a negatively charged surface. There is a positive surface formed by R250 and R251 in the JM region and a hydrophobic patch formed by the three hydrophobic residues including L253, I257 and W258 (Fig. 5).

**Figure 6:**
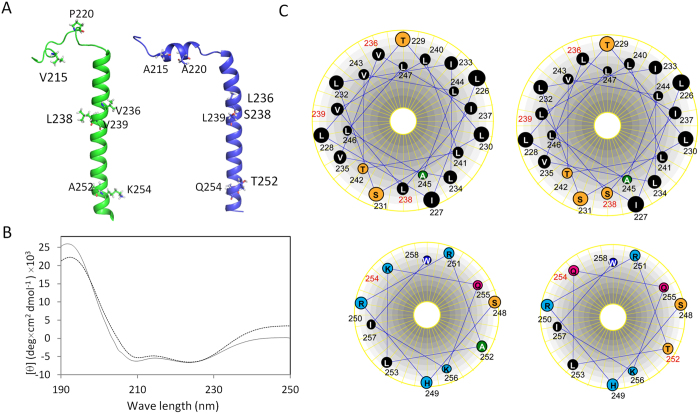
Structural comparison between mEpoR and hEpoR. (**A**) Structures of the hEpoR and mEpoR. Left panel is the structure of the hEpoR (PDB id 2MV6). The right panel is the structure of the mEpoR. The different residues between these two receptors are shown in sticks and labeled. (**B**) CD analysis of the mEpoR and hEpoR. The CD spectra of both mouse and human (dotted line) EpoRs were collected and shown. (**C**) Helix-wheel analysis of TMDs and JM regions of the human and mouse EpoRs. The upper panel is the TMDs from human (left) and mouse (right) EpoR and the lower panel is the JM regions from corresponding receptors. The sequence numbers for the different residues between human and mouse EpoR are shown in red.

The structure of the mEpoR is similar to that of the human EpoR except that there is a short helix at the N-terminal region (Fig. 6A). Further CD spectra of the mouse and human EpoRs showed that these two proteins had slightly different spectra and mEpoR exhibited slightly lower maximum at 208 nm (Fig. 6B) , suggesting that the helical content in mEpoR is slightly more than hEpoR. Helix wheel presentation showed that the sequence difference at position 238 in the TMD might explain the functional difference observed between mEpoR and hEpoR. S238 in mEpoR (L238 in hEpoR) forms a hydrophilic patch with S231 and T242 (Fig. 6C). This patch may favor TMD-TMD dimerization because it behaves like a small-XXX-small motif that is commonly used in TMD-TMD interactions^15^. The presence of this hydrophilic patch may explain why mEpoR has a higher dimerization affinity than hEpoR. For the JM region, the amino acid difference at position 254 did not alter the position of the three conserved hydrophobic residues including L253, I257 and W258 in both receptors, which still can form a hydrophobic patch that is important for the receptor function (Fig. 6C).

## Discussion

The signal transduction through EpoR includes the movement of its TMD. To investigate the mechanism, we solved the solution structure of the mEpoR in DPC micelles. DPC has been used in many structural studies of membrane proteins due to its close structure to the cell membrane^16^. The DPC micelles might not be an ideal system for the mEpoR to form dimers because only a minor population of dimer species was observed in the sample^17^ (Fig. S1). It was also shown that hEpoR could form dimer or trimers in different micelles, suggesting that the membrane mimicking system affects receptor dimerization^13^. As the length of the mEpoR used in this study is short, the structural information of the mEpoR obtained in micelles will be close to the physiological conditions^18^. Previous studies on GHR and full-length cytochrome P450 have suggested that the structures of the TMDs were not altered during signal transduction^10^,^19^. A study on the TMD of the TpoR-a homology of EpoR showed that it could form three stable conformations. Rotation of the TMD might also be one of the mechanisms during receptor activation^20^. The rigidity of the mEpoR TMD also suggested that there might not be any conformational changes in the TMD during signal transduction.

There is only seven amino acids difference between the mEpoR and hEpoR that were used in structural studies^13^. Both receptors have similar structures and dynamics (Fig. 6). The residues preceding the TMD are demonstrated to be dynamic for both mEpoR and hEpoR (Fig. 4). As predicted from a previous study, TMD and the JM region formed an extended helix^8^. Dynamic study show the C-terminal JM regions both receptors containing residues L253 to W258 are stable, but not as rigid as the TMDs in DPC micelles (Fig. 4). Although the JM region contains several conserved hydrophobic residues that are important for signal transduction, this region was shown to be exposed to the solvent (Fig. 4). These three hydrophobic residues might bind JAK2 under certain conditions. Structural difference was also observed between the human and mouse receptors (Fig. 6). For the mEpoR, there is a short helix preceding the TMD (Fig. 2), which affected the length between the extracellular domain and the TMD. Having a short helix will shorten the length of the linker region, which might be one of the reasons that mouse EpoR has a stronger dimerization affinity than human EpoR. Although TMDs of both mEpoR and hEpoR adopt similar structures, there are three different residues present in this domain (Fig. 1). In mEpoR residue 231 is a serine (S231) in place of a leucine, which forms a hydrophilic patch with other residues including S238 and T242. This patch can favor TMD-TMD interactions (Fig. 6C), which might make mEpoR have a stronger dimerization affinity than hEpoR.

The TMD of the EpoR was shown to be essential for the dimerization of the entire dimer^7^. By replacing the extracellular domain of the EpoR with a dimeric coiled coil, Seubert *et al.* have identified three different TM conformations representing fully active, partially active and inactive receptors^21^. Residues including S231, S238, T242 and S248 were predicted to be important for dimerization^21^. In a double mutation study, Kubatzky *et al.* showed that residues L240 and L241 are important for EpoR TMD dimerization^22^. In their study, it was shown that the dimerization mechanisms might be different for GHR and EpoR^22^. We also investigated the EpoR dimerization in DPC micelles. The EpoR dimeric species was observed when the mEpoR was reconstituted in DPC micelles in a cross-linking study (Fig. S1). Several residues exhibited line broadening in the ^1^H-^15^N-HSQC spectra at different protein to DPC ratios suggesting that mEpoR can form dimers in DPC micelles (Fig. S2). A left-hand dimer structure of mEpoR can be built based on few inter-molecular NOEs observed from residues including S231, S238 and T241 (Fig. S3). It has been noted that the number of the inter-molecular NOEs observed in this system could not generate high resolution structures. This might arise from the fact that DPC is not an ideal system for mEpoR to form dimers because the mEpoR dimer species were low even at a high protein to DPC ratio and in the presence of a cross linker (Fig. S1). Although detergent micelles is suitable for solution NMR structural study of membrane proteins due to the size of protein-detergent complex, detergents tend to destabilize protein-protein or protein-lipid interactions. Bicelle systems such as isotropic bicelles might be a suitable system for studying EpoR dimer structures because this system is able to sustain structure and function of membrane proteins containing both TMD and large water soluble regions^23^,^24^,^25^. Further structural study of this construct in other membrane systems or a new construct containing the extracellular domain and TMD using both solution and solid states NMR^26^ will provide more information to understand signal transduction through the EpoR.

In summary, we solved the solution structure of the TMD the JM regions of mEpoR in DPC micelles. The mEpoR showed similar structure to hEpoR with the exception that the N-terminal region preceding the TMD contains a short helix. The N-terminal region of the EpoR localized between the extracellular domain and the TMD is flexible. The TMD forms a rigid helix in DPC micelles. The C-terminal JM region is helical and stable in solution. The TMD of mEpoR contains a hydrophobic patch formed by residues S231, S238 and T242 and this patch may be important for dimerization.

## Experimental Procedures

### Materials

The cDNA encoding residues S212-P259 of the mEpoR were synthesized by Genscript. Expression vector such as pET-29b plasmid was purchased from Merck. The SDS-PAGE system was purchased from Invitrogen. Protein sample loading dye, molecular weight standards were purchased from Bio-rad. Bl21 (DE3) competent cells were purchased from StrataGene. β-D-1-thiogalactopyranoside (IPTG), Dithiothreitol (DTT) and detergents including dodecylphosphocholine (DPC), deuterated DPC (D-DPC) were purchased from Anatrace or Avanti. The ^15^NH_4_Cl, ^13^C-glucose and D_2_O were purchased from Cambridge Isotope Laboratories. All other chemicals were purchased from Sigma.

### Expression and purification of the EpoR

The mouse EpoRs were cloned into the NdeI and XhoI sites of pET29b and the resulting plasmid pET29-mEpoR encodes a protein sequence containing S212-P259 of mouse EpoR (mEpoR) with six histidine residues (HHHHHH) at its C-terminus. The EpoR was expressed and purified as described previously^13^,^27^. To prepare for a sample with low DPC to protein ratio, mEpoR was eluted with an elution buffer containing 20 mM sodium phosphate, 300 mM imidazole and 5 mM DPC. Purified fractions with low DPC to protein ratio were collected and was further purified through a gel filtration chromatography and concentrated for further studies.

### NMR experiments and structure determination

Uniformly ^13^C/^15^N-labeled mEpoR was prepared using the aforementioned method. Sample was concentrated to 0.5 mM in a buffer containing 20 mM sodium phosphate, pH 6.5, 1 mM DTT, 200 mM D-DPC and 10% D_2_O. NMR spectra were recorded at 313 K on a Bruker Avance 600 or Avance 700 spectrometer with a cryogenic triple resonance probe. Data acquired were processed with NMRPipe^28^ and analyzed using NMRView^29^. Secondary structure was identified by analysis of ^13^C secondary chemical shifts^30^ and TALOS+^31^. Distance constraints were collected from a 3D ^15^N-edited NOESY (mixing time = 100 ms) experiment under the aforementioned conditions. The NOE connection was plotted using CYANA^32^. The hEpoR structure determination was carried out using XPLOR-NIH with python interface^33^,^34^,^35^. The backbone dihedral angles restraints were generated using TALOS+ based on the backbone chemical shifts^31^. The nuclear Overhauser effect (NOE) peaks were picked manually from a 3D ^15^N-edited NOESY and calibrated using NMRView^29^. Structure determination was carried out using a randomized template. Simulated annealing was performed and energy minimization were carried out as previously described^36^.H-D exchange experiment was carried out using the method described^37^. *T*_1_, *T*_2_ and hetNOE experiments^38^ were measured at 313K on a Bruker Avance II 600 MHz spectrometer. For *T*_1_ measurement, the relaxation delays of 10, 50, 100, 200, 400, 800, 1200, 1400, 1600 and 1800 ms were recorded. For *T*_2_ measurement, the data was acquired with delays of 16.9, 34, 51, 68, 85, 102, 119, 136 and 153 ms. The hetNOE was obtained using two datasets that were collected with and without initial proton saturation for a period of 3 s 39. Protein structures were analyzed by using PROCHECK-NMR^40^. The relaxation was further analyzed using model-free^41^,^42^.

### Circular dichroism (CD) Spectroscopy

Far-UV CD spectrum of the peptide at a concentration of 20 μM was analyzed in a buffer containing 20 mM sodium phosphate, pH6.5 and 0.5% dodecylphosphocholine (DPC). The instrument was referenced against a cuvette containing buffer without EpoR. The CD spectra were recorded at 25 °C and 40 °C.

### Bioinformatics analysis of the mEpoR sequence

Predicting amino acid exposure based on the amino acid sequence was carried out using half-sphere exposure (HES) method^14^. The protein sequence was analyzed using the web server (http://sunflower.kuicr.kyoto-u.ac.jp/~sjn/hse/). The obtained HES-up, HSP-down, and contact number (CN) were plotted against residue number.

### Fluorescence spectroscopy

The fluorescence measurement was conducted as previously described^43^,^44^. The fluorescence emission spectra were measured in a 96-well plate. Purified hEpoR was prepared in 50 μM in a buffer that contained 20 mM sodium phosphate, pH6.5 and 40 mM DPC. Samples with 100 μl volume were subjected to analysis. Excitation wavelength was 280 nm and the emission was scanned from 305 to 400 nm. Experiment was carried out at 37 °C.

## Additional Information

**Accession numbers**: NMR assignments for the mEpoR have been deposited in the BioMagResBank (http://www.bmrb.wisc.edu) under ID code 25396. The structure of mEpoR has been deposited in the PDB under ID code 2MXB.

**How to cite this article**: Li, Q. *et al.* Solution structure of the transmembrane domain of the mouse erythropoietin receptor in detergent micelles. *Sci. Rep.* **5**, 13586; doi: 10.1038/srep13586 (2015).

## Supplementary Material

Supplementary Information

## Figures and Tables

**Table 1:** Summary of the 20 structures of mEpoR in DPC micelles.

Number of unambiguous NOEs	
Short range (|i–j|≤1)	222
Medium-range (1 <|i–j|<5)	53
Long-range (|*i*–*j|*>4)	0
Number of dihedral angle constraints	90
Number of hydrogen-bond restraints	21
Number of restraint violations^a^	
Total number of restraint violations >0.5 Å	0
Total number of dihedral angle constraints >5°	0
Ramachandran plot statistics^b^ (%)	
Residues in most favoured regions	89.7
Residues in additionally allowed regions	9.6
Residues in generously allowed regions	0.8
Residues in disallowed regions	0
Average RMSD to mean (Å)	
Backbone (residues 226–258)	0.60 ± 0.21 Å
Heavy atoms (residues 226–258)	1.48 ± 0.23 Å

^a^There are no distance violations greater than 0.5 Å or dihedral angle violations greater than 5°.

^b^The Ramachandran plot was obtained using PROCHECK-NMR^40^ based on the conformer with lowest energy.

## References

[b1] Palis J. Primitive and definitive erythropoiesis in mammals. Front Physiol 5, 3 (2014). 24478716 10.3389/fphys.2014.00003 PMC3904103

[b2] Richmond T. D., Chohan M. & Barber D. L. Turning cells red: signal transduction mediated by erythropoietin. Trends Cell Biol 15, 146–155 (2005). 15752978 10.1016/j.tcb.2005.01.007

[b3] Witthuhn B. A. *et al.* JAK2 associates with the erythropoietin receptor and is tyrosine phosphorylated and activated following stimulation with erythropoietin. Cell 74, 227–236 (1993). 8343951 10.1016/0092-8674(93)90414-l

[b4] D’Andrea A. D., Lodish H. F. & Wong G. G. Expression cloning of the murine erythropoietin receptor. Cell 57, 277–285 (1989). 2539263 10.1016/0092-8674(89)90965-3

[b5] Livnah O. *et al.* Crystallographic evidence for preformed dimers of erythropoietin receptor before ligand activation. Science 283, 987–990 (1999). 9974392 10.1126/science.283.5404.987

[b6] Livnah O. *et al.* An antagonist peptide-EPO receptor complex suggests that receptor dimerization is not sufficient for activation. Nat Struct Biol 5, 993–1004 (1998). 9808045 10.1038/2965

[b7] Constantinescu S. N. *et al.* Ligand-independent oligomerization of cell-surface erythropoietin receptor is mediated by the transmembrane domain. Proc Natl Acad Sci USA 98, 4379–4384 (2001). 11296286 10.1073/pnas.081069198 PMC31843

[b8] Constantinescu S. N., Huang L. J., Nam H. & Lodish H. F. The erythropoietin receptor cytosolic juxtamembrane domain contains an essential, precisely oriented, hydrophobic motif. Mol Cell 7, 377–385 (2001). 11239466 10.1016/s1097-2765(01)00185-x

[b9] Pelletier S. *et al.* Two domains of the erythropoietin receptor are sufficient for Jak2 binding/activation and function. Mol Cell Biol 26, 8527–8538 (2006). 16982687 10.1128/MCB.01035-06 PMC1636781

[b10] Brooks A. J. *et al.* Mechanism of activation of protein kinase JAK2 by the growth hormone receptor. Science 344, 1249783 (2014). 24833397 10.1126/science.1249783

[b11] Waters M. J., Brooks A. J. & Chhabra Y. A new mechanism for growth hormone receptor activation of JAK2, and implications for related cytokine receptors. JAKSTAT 3, e29569 (2014). 25101218 10.4161/jkst.29569 PMC4119067

[b12] Ebie A. Z. & Fleming K. G. Dimerization of the erythropoietin receptor transmembrane domain in micelles. J Mol Biol 366, 517–524 (2007). 17173930 10.1016/j.jmb.2006.11.035

[b13] Li Q., Wong Y. L., Huang Q. & Kang C. Structural insight into the transmembrane domain and the juxtamembrane region of the erythropoietin receptor in micelles. Biophys J 107, 2325–2336 (2014). 25418301 10.1016/j.bpj.2014.10.013 PMC4241451

[b14] Song J., Tan H., Takemoto K. & Akutsu T. HSEpred: predict half-sphere exposure from protein sequences. Bioinformatics 24, 1489–1497 (2008). 18467349 10.1093/bioinformatics/btn222

[b15] Fink A., Sal-Man N., Gerber D. & Shai Y. Transmembrane domains interactions within the membrane milieu: Principles, advances and challenges. Biochimica et Biophysica Acta (BBA) - Biomembranes 1818, 974–983 (2012). 22155642 10.1016/j.bbamem.2011.11.029

[b16] Kang C. & Li Q. Solution NMR study of integral membrane proteins. Curr Opin Chem Biol 15, 560–569 (2011). 21684799 10.1016/j.cbpa.2011.05.025

[b17] Zhang M. *et al.* Effects of Membrane Mimetics on Cytochrome P450-Cytochrome b5 Interactions Characterized by NMR Spectroscopy. J Biol Chem 290, 12705–12718 (2015). 25795780 10.1074/jbc.M114.597096 PMC4432288

[b18] Franzin C. M., Teriete P. & Marassi F. M. Structural similarity of a membrane protein in micelles and membranes. J Am Chem Soc 129, 8078–8079 (2007). 17567018 10.1021/ja0728371 PMC2518691

[b19] Yamamoto K. *et al.* Probing the transmembrane structure and topology of microsomal cytochrome-p450 by solid-state NMR on temperature-resistant bicelles. Sci Rep 3, 2556 (2013). 23989972 10.1038/srep02556 PMC3757361

[b20] Matthews E. E. *et al.* Thrombopoietin receptor activation: transmembrane helix dimerization, rotation, and allosteric modulation. FASEB J 25, 2234–2244 (2011). 21402716 10.1096/fj.10-178673 PMC3114528

[b21] Seubert N. *et al.* Active and inactive orientations of the transmembrane and cytosolic domains of the erythropoietin receptor dimer. Mol Cell 12, 1239–1250 (2003). 14636581 10.1016/s1097-2765(03)00389-7

[b22] Kubatzky K. F. *et al.* Self assembly of the transmembrane domain promotes signal transduction through the erythropoietin receptor. Curr Biol 11, 110–115 (2001). 11231127 10.1016/s0960-9822(01)00018-5

[b23] Ahuja S. *et al.* A model of the membrane-bound cytochrome b5-cytochrome P450 complex from NMR and mutagenesis data. J Biol Chem 288, 22080–22095 (2013). 23709268 10.1074/jbc.M112.448225 PMC3724662

[b24] Kang C. *et al.* Functional delivery of a membrane protein into oocyte membranes using bicelles. Biochemistry 49, 653–655 (2010). 20044833 10.1021/bi902155t PMC2811756

[b25] Dürr U. H. N., Gildenberg M. & Ramamoorthy A. The Magic of Bicelles Lights Up Membrane Protein Structure. Chemical Reviews 112, 6054–6074 (2012). 22920148 10.1021/cr300061w PMC3497859

[b26] Yamamoto K. *et al.* Temperature-Resistant Bicelles for Structural Studies by Solid-State NMR Spectroscopy. Langmuir 31, 1496–1504 (2015). 25565453 10.1021/la5043876

[b27] Li Q., Wong Y. L. & Kang C. Solution structure of the transmembrane domain of the insulin receptor in detergent micelles. Biochim Biophys Acta 1838, 1313–1321 (2014). 24440425 10.1016/j.bbamem.2014.01.005

[b28] Delaglio F. *et al.* NMRPipe: a multidimensional spectral processing system based on UNIX pipes. J Biomol NMR 6, 277–293 (1995). 8520220 10.1007/BF00197809

[b29] Johnson B. A. Using NMRView to visualize and analyze the NMR spectra of macromolecules. Methods Mol Biol 278, 313–352 (2004). 15318002 10.1385/1-59259-809-9:313

[b30] Wishart D. S., Sykes B. D. & Richards F. M. The chemical shift index: a fast and simple method for the assignment of protein secondary structure through NMR spectroscopy. Biochemistry 31, 1647–1651 (1992). 1737021 10.1021/bi00121a010

[b31] Shen Y., Delaglio F., Cornilescu G. & Bax A. TALOS+: a hybrid method for predicting protein backbone torsion angles from NMR chemical shifts. J Biomol NMR 44, 213–223 (2009). 19548092 10.1007/s10858-009-9333-z PMC2726990

[b32] Guntert P. Automated NMR structure calculation with CYANA. Methods Mol Biol 278, 353–378 (2004). 15318003 10.1385/1-59259-809-9:353

[b33] Schwieters C. D., Kuszewski J. J., Tjandra N. & Clore G. M. The Xplor-NIH N. M. R. molecular structure determination package. J Magn Reson 160, 65–73 (2003). 12565051 10.1016/s1090-7807(02)00014-9

[b34] Banci L. *et al.* Paramagnetism-based restraints for Xplor-NIH. J Biomol NMR 28, 249–261 (2004). 14752258 10.1023/B:JNMR.0000013703.30623.f7

[b35] Charles D. Schwieters J. J. K. & Marius Clore G. Using Xplor–NIH for NMR molecular structure determination. Progress in Nuclear Magnetic Resonance Spectroscopy 48, 47–2002 (2006).

[b36] Kang C. *et al.* Structure of KCNE1 and implications for how it modulates the KCNQ1 potassium channel. Biochemistry 47, 7999–8006 (2008). 18611041 10.1021/bi800875q PMC2580054

[b37] Veglia G., Carolina Zeri A., Ma C. & Opella S. J. Deuterium/Hydrogen Exchange Factors Measured by Solution Nuclear Magnetic Resonance Spectroscopy as Indicators of the Structure and Topology of Membrane Proteins. Biophysical Journal 82, 2176–2183 (2002). 11916873 10.1016/s0006-3495(02)75564-1 PMC1302011

[b38] Kay L. E., Torchia D. A. & Bax A. Backbone dynamics of proteins as studied by 15N inverse detected heteronuclear NMR spectroscopy: application to staphylococcal nuclease. Biochemistry 28, 8972–8979 (1989). 2690953 10.1021/bi00449a003

[b39] Gayen S. *et al.* An NMR study of the N-terminal domain of wild-type hERG and a T65P trafficking deficient hERG mutant. Proteins 79, 2557–2565 (2011). 21661061 10.1002/prot.23089

[b40] Laskowski R. A. *et al.* AQUA and PROCHECK-NMR: programs for checking the quality of protein structures solved by NMR. J Biomol NMR 8, 477–486 (1996). 9008363 10.1007/BF00228148

[b41] Cole R. & Loria J. P. FAST-Modelfree: a program for rapid automated analysis of solution NMR spin-relaxation data. J Biomol NMR 26, 203–213 (2003). 12766418 10.1023/a:1023808801134

[b42] Chen J., Brooks C. L. 3rd & Wright P. E. Model-free analysis of protein dynamics: assessment of accuracy and model selection protocols based on molecular dynamics simulation. J Biomol NMR 29, 243–257 (2004). 15213423 10.1023/B:JNMR.0000032504.70912.58

[b43] Conner M. *et al.* Functional and biophysical analysis of the C-terminus of the CGRP-receptor; a family B GPCR. Biochemistry 47, 8434–8444 (2008). 18636754 10.1021/bi8004126

[b44] Gayen S., Li Q., Kim Y. M. & Kang C. Structure of the C-terminal region of the Frizzled receptor 1 in detergent micelles. Molecules 18, 8579–8590 (2013). 23881048 10.3390/molecules18078579 PMC6269726

