# Supplementary material for: Solution structure of the transmembrane domain of the mouse erythropoietin receptor in detergent micelles
Source: Sci Rep. 2015 Aug 28;5:13586. doi: 10.1038/srep13586 (PMC4551963; doi:10.1038/srep13586)
Supplement: Supplementary Information [file srep13586-s1.pdf]

Supplementary Information

Solution structure of the transmembrane domain of the mouse erythropoietin receptor in detergent micelles

Short title: NMR study of the mouse erythropoietin receptor

Qingxin Li[1], Ying Lei Wong[2], Michelle Yueqi Lee[2], Yan Li[2], CongBao Kang[2,*]

[1]Institute of Chemical & Engineering Sciences, Agency for Science, Technology and Research (A*STAR), Singapore, Singapore

[2]Experimental Therapeutics Centre, Agency for Science, Technology and Research (A*STAR), Singapore, 138669 Singapore

To whom correspondence should be addressed: CongBao Kang, 31 Biopolis Way Nanos, #03-01, Singapore. Tel: 65-64070602; Fax: 65-64788768; Email: cbkang@etc.a-star.edu.sg

Author contributions: C.K. and Q.L. conceived the study. Q.L., Y.L.W., M.Y.L., Y. L. and C.K. performed the experiments. Q.L. and C.K. wrote the manuscript.

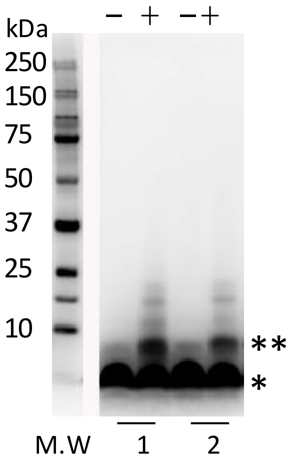

Figure S1. Cross-linking study of the mEpoR with different DPC to protein ratios. 1 is the sample with a DPC to protein ratio of 150 and 2 is the sample with a DPC to protein ratio of 300. Cross-linking of EpoR samples using glutaraldehyde (GA) was carried out using the same method as previously described[1]. Briefly, purified mEpoR from *E. coli* were buffer-exchanged to the cross-linking buffer that contained 20 mM Sodium phosphate, pH6.5, 0.1 mM DTT and 20 mM detergent. The protein concentration was diluted to 50 μM to a final volume of 50 μl. GA cross linker was added into protein solution to 16 mM and the mixture was incubated at room temperature for 10 min followed by SDS-PAGE and Western blot using an anti-his antibody. The monomeric and dimeric species are labelled with "*" and "**", respectively.

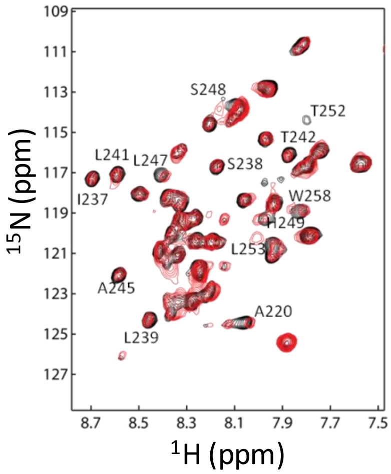

Figure S2. ^{1}H-^{15}N-HSQC spectra of the mEpoR at different DPC to protein ratios. The two samples used for cross-linking study were also subjected to analysis by NMR. The spectra of samples at low (red) and high (black) DPC to protein ratios are shown. The two samples exhibited similar spectra with the exception that several residues showed line broadening and few residues showed chemical shift perturbation. The affect residues include I237, S238, L239, L241, T242, A245 and L247 from the TMD and residues S248, H249, T252, L253 and W258 from the C-terminal JM region. These affected residues might be important for or affected by TMD dimerization

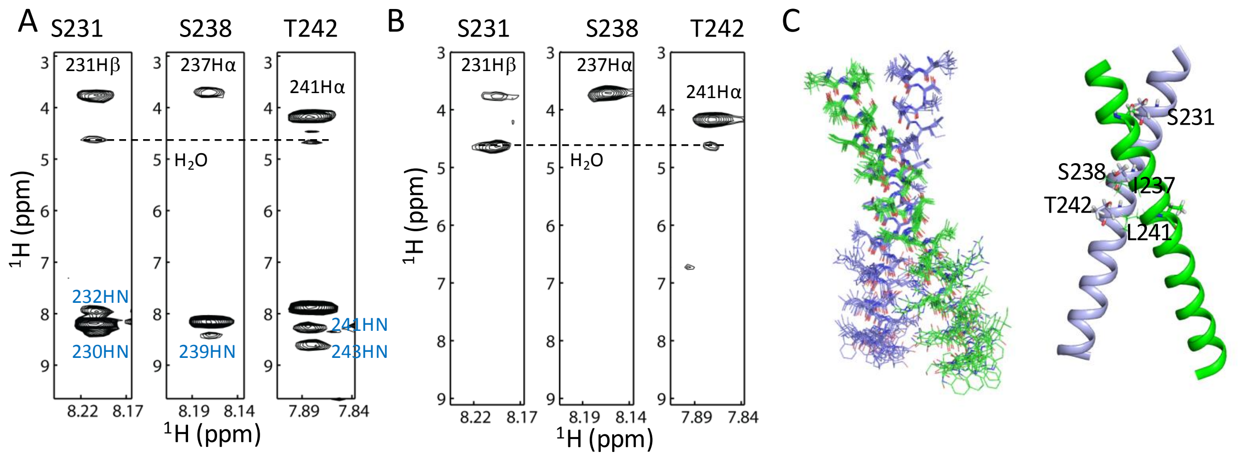

Figure S3. Inter-molecular NOE analysis of the mEpoR. A. Selected trip of ^{15}N-edited NOESY obtained from a sample composed of a mixture of ^{2}H/^{15}N-labled and un-labeled mEpoR in a 1:1 ratio in D-DPC micelles. Intra-molecular amide-amide NOEs are labeled and shown in blue. Residue names are labeled on the top of the spectra. B.^{15}N-filtered NOE analysis. Selected strip of F1-^{13}C/^{15}N-filtered F2-^{15}N-edited NOESY experiment obtained from a sample composed of a mixture of ^{13}C/^{15}N-labled and un-labeled mEpoR in a 1:1 ratio in D-DPC micelles. C. Dimer structural model of the mEpoR. Left panel is the ensemble of 10 superimposed lowest energy structure of the mEpoR dimer using XPLOR-NIH [2]. This model was generated using the few intermolecular NOEs observed. For clarity, the N-terminus of the construct is not shown. Right panel is the ribbon representation of the lowest energy dimer of the mEpoR. The two monomers are shown in light blue and green, respectively. Residues having inter-molecular NOE observed are labeled.

References

1. Vinogradova O*, et al.* Escherichia coli diacylglycerol kinase: a case study in the application of solution NMR methods to an integral membrane protein. *Biophys J* **72**, 2688-2701 (1997).

2. Schwieters CD, Kuszewski JJ, Tjandra N, Clore GM. The Xplor-NIH NMR molecular structure determination package. *J Magn Reson* **160**, 65-73 (2003).
